# Surgery

**Published:** 1894-01

**Authors:** 


### SURGERY.

EDITED BY DR. F. W. McRAE.

### ON THE TREATMENT OF EMPYEMA.

In the *British Medical Journal* of July 22, 1893, Chapman gives his views on the treatment of empyema. He has operated with success in seventeen cases. The spot for incision should be determined by the peculiarities of the case. As there is always a preliminary puncture over the maximum seat of dullness, the incision should be also over this region, to include the puncture. Both should be made as far back as possible. In practice he finds the spot situated, most often, at about the upper edge of the ninth and tenth ribs, in a line with the angle of the scapula. By two strokes of the knife the chest is opened, and two fingers inserted as far as possible. He advises the insertion of a finger once a week during the subsequent dressings, even if all appears to be going on well. Counter openings have never been found necessary. Resection of a rib he believes unnecessary, but in an extreme case a simple division of one is performed. The only satisfactory drainage is a simple one, three or four inches long, with thick walls. It should have attached to it a shield, two and a half inches square, and should be held in position by four tapes, two around the body and two over the opposite shoulder. The discharge is received in salembroth wool.—*University Medical Magazine.*

---

### The Etiology, Diagnosis and Treatment of Ulceration of the Rectum.

Dr. Joseph M. Mathews, of Louisville, read a paper on this subject before the Mississippi Valley Medical Association.

The author dealt with this subject in a general way. As a matter of convenience, he classified these ulcers under four heads: Benign, malignant, tubercular and specific. He said to this division there might be a valid objection based upon correct pathological grounds. For instance, in this classification he makes the term malignant synonymous with cancer, and yet the tubercular ulcer may in truth be malignant without assuming the characteristics of cancer. Again, some writers would have us believe that a tuberculous patient is closely akin at least to a syphilitic one, or vice versa. Benign ulceration is not so frequently found in the rectum as is supposed. Whenever the author meets with a well defined ulceraion existing in the rectum, he immediately begins to suspect some special diathesis. It is only when the facility of defection is interfered with that any danger is to be feared. The lumen of this portion of the gut is not narrowed by any ordinary causes. For these and other causes Dr. Mathews has long since been forced to believe that such ascribed causes as pregnancy, dysentery, etc., were not great factors in producing ulceration of the rectum. The speaker then dwelt upon malignant ulceration of the rectum, saying that the rectum is a favorite seat for cancer. Many times the disease is overlooked entirely or diagnosticated as some other affection. It has occurred to him several times to have had patients referred to him for some trivial rectal trouble and found caneer instead. The more he practices the more he is convinced that syphilis is responsible for fully one-half of the cases of severe ulceration of the rectum.

The treatment must in every individual case depend upon the known character of it. We cannot treat a benign ulcer as we would a malignant one, nor a tubercular ulcer as a specific one, nor the last named as the first named. Benign growths of the rectum require local applications. Malignant, extirpation ; tuberculous, the curette ; syphilitic, antisyphilitic and local medication, extirpation, and colotomy.—*Indiana Medical Journal.*

---

### BUBOES AND THEIR TREATMENT.

Dr. J. G. Sherrill, of Louisville, Ky. (*New York Medical Journal*), has adopted the following method of treatment, which he says was first brought to his notice by Dr. Vance of that city. He thinks it possesses so many advantages over the other plans of treatment that it should become more generally adopted. Instead of wasting time trying to abort the inflammation, and after watching the case for two or three days, if the patient still suffers much pain and inconvenience, he resorts to the immediate removal of all the inflamed glands. A free incision over the inflamed tissue down to the glandular structure will reveal the inflamed gland lying loosely in its capsule. Very little difficulty will be experienced in its removal. The hemorrhage should be controlled and the wound irrigated with hot water containing some antiseptic ; if preferred, dried and sprinkled with iodoform ; then packed with iodoform gauze and covered with a compress fixed by a spica. The dressing should be changed on the second day, and at longer intervals after that time. The advantages afforded by this method are :

1. A complete and rapid recovery—at the very longest, in two weeks.

2. Relief of the patient from a number of weeks suffering and inconvenience, as it is possible for him to be out in three days, and the pain is instantly relieved.

3. The almost certain prevention of a virulent sore, and this of necessity excludes the possibility of the wound being attacked by phagedena.

4. The absence of danger or difficulty in carrying out the treatment.

5. The relief to the surgeon from the well-worn phrase, "Doctor, I am no better." Hearing this every day or so for a number of weeks grows very tiresome.—*Columbus Med. Jour.*

---

### SUBACUTE MASTITIS AND CANCER OF THE BREAST.

In *L'Union Médicale*, March 21, 1893, Reclus gives a valuable clinical lecture on the differential diagnosis of subacute mastitis and carcinoma. Subacute mastitis has for its causes, usually, traumatism and lactation. The tumefaction of the breast, the indolence of its course, the thickening and induration of the skin, the retraction of the nipple, the absence of fluctuation—these signs all belong particularly to cancer, but they may likewise accompany a subacute mastitis. While lactation predisposes to mastitis it does not preclude cancer. While pain is in favor of mastitis, still it is far from being decisive. The same is the case with glandular involvement; in mastitis it is apt to be more violent and rapid. The various symptoms taken individually are not at all reliable, and it is only by considering all the symptoms of the case that its nature can be correctly diagnosed. Oftentimes a few days or at most a few weeks will resolve all doubts. Care should be taken not to amputate a breast affected only with mastitis, nor, as occurred in one of our cases, open a fluctuating cancerous nodule for a simple abscess. One should, above all, not forget that true pathognomonic signs do not exist, but that it is necessary to examine all the symptoms and especially the mode of onset and development, and whether or not it is connected with lactation.—*Univ. Med. Jour.*

---

### GANGLION.

Dr. H. M. Jordan reports twenty-five cases (*Lancet*, July 29, 1893.)

He defines the word ganglion as meaning "a small cyst in connection with the sheath of a tendon." They vary from a pin's head to a walnut in size, the capsule of the sac consisting of fibrous tissue lined internally with synovial membrane. The contents vary in color and consistence from a clear transparent fluid to a deep yellow or pink viscid substance—the usual condition being a faint pink color and a consistence similar to ordinary synovial fluid. In its growth a ganglion may press on cutaneous nerves, causing pain referred to area of distribution. Its most usual situation is in connection with the tendons of the extensor communis digitorum at the back of the wrist; and it is also found on the dorsum of foot about the tendons of the extendor longus digitorum. Occasionally, also, one is found of small size firmly attached to the flexor tendons on the proximal phalanges of the fingers.

After enumerating the explanations of various authorities of the causation of the lesion, the author supports the view that it arises from a hernial protrusion of the synovial membrane through a slit or rent in the fibrous sheath, the neck being compressed by the tightness in the rent of the sheath; a plastic inflammation is set up obliterating the neck, and the sac is thus shut off from the rest of the synovial cavity. His reasons for this view are: (1) The frequency of the affection in persons who occasionally have hard manual work to perform, such as charwomen who have "wringing" to do. (2) The sudden and painful onset succeeding some exceptionally hard work, as noticed in the cases quoted. (3) The results of careful dissection, showing that the fibrous capsule of the sac is of new formation from areolar tissue and not a mere expansion of the fibrous sheath.

The various methods hitherto adopted for treatment are mentioned at some length, namely: "Rupture by pressure of thumbs, or by sharp blow. (2) Electrolysis. (3) Puncture and scarification, a valvular opening being made into the cyst, the contents squeezed out, and the interior thoroughly scarified, followed by the use of splints and gentle pressure. (4) Seton; carbolized silk, gut or horse-hair being used. (5) Incision into cyst and then allowing it to granulate up from the bottom. (6) Excision by carefully dissecting out the unopened cyst, and then cutting it away from the sheath.

These methods are objected to for various reasons—recurrence, suppuration in sheath, matting of tendons together, unsightly scars.

It is by a combined method of aspiration and injection that the author has treated his cases. The object aimed at is to cause inflammation of the synovial tunic, which becomes covered with layers of plastic lymph, and this lymph subsequently organizing, the synovial membrane as a secreting surface is destroyed, and by keeping the walls of sac in apposition the cavity is obliterated.

The operation is thus described: The part being scrupulously cleansed, a large hypodermic syringe, with a wide-bored needle which is screwed on, after being carefully asepticized, was thrust into the ganglion and the contents withdrawn. Often there was considerable difficulty in getting these to flow through the needle, in which event firm pressure on the tumor synchronous with the aspiration overcame the difficulty. The syringe being unscrewed, with the needle left in position, a piece of lint wrung out of a 5 per cent. carbolic acid solution, was placed at once over the needle. The syringe, emptied and then filled with Morton's fluid, was rescrewed on to the needle and the sac partly filled. On withdrawing the needle the "tumor" was freely manipulated to insure the fluid reaching every part of the interior. A splint reaching to the finger tips was applied, and a thick, tightly-folded pad of lint firmly strapped over the tumor, and the arm suspended in a sling. —*Med. Chronicle.*

---

### CHLORIDE OF AMMONIA IN THE TREATMENT OF CYSTITIS.

Dr. G. Corrie (*La Semaine Médicale, No. 58, 1893*) finds the chloride of ammonium to be superior to all other means in the treatment of the various varieties of cystitis. Under the influence of this drug, in doses of one to two grammes (15 to 30 grains), he has seen the urine clear up, and all the morbid symptoms disappear with surprising rapidity. It may be given in still larger doses, and in no case did it produce any gastric disturbance or disagreeable symptoms.—*Lancet-Clinic.*

